# An analysis of retrospective and repeat prospective reports of adverse childhood experiences from the South African Birth to Twenty Plus cohort

**DOI:** 10.1371/journal.pone.0181522

**Published:** 2017-07-26

**Authors:** Sara N. Naicker, Shane A. Norris, Musawenkosi Mabaso, Linda M. Richter

**Affiliations:** 1 Human & Social Development Programme, Human Sciences Research Council, KwaZulu-Natal, South Africa; 2 MRC/Wits Developmental Pathways for Health Research Unit, University of the Witwatersrand, Johannesburg, South Africa; 3 HIV/AIDS, STIs & TB Programme, Human Sciences Research Council, KwaZulu-Natal, South Africa; 4 DST-NRF Centre of Excellence in Human Development, University of the Witwatersrand, Johannesburg, South Africa; Stellenbosch University, SOUTH AFRICA

## Abstract

Most studies rely on cross-sectional retrospective reports from adult samples to collect information about adverse childhood experiences (ACEs) to examine relationships with adult outcomes. The problems associated with these reports have long been debated, with only a few studies determining their reliability and validity and fewer still reaching consensus on the matter. This paper uses repeat prospective and retrospective reports of adverse childhood experiences from two respondent sources in the South African Birth to Twenty Plus (Bt20+) cohort to explore agreement and concordance in the prospective reporting of ACEs by caregivers and respective children as adolescents and then as young adults. The findings demonstrate little overall agreement between prospective and retrospective accounts of childhood experiences, with 80% of kappa values below the moderate agreement cutoff (*k* = .41). The highest levels of agreement were found between prospective and retrospective reporting on parental and household death (kappas ranging from .519 to .944). Comparisons between prospective caregiver reports and retrospective young adult reports yielded high concordance rates on sexual and physical abuse and exposure to intimate partner violence (91.0%, 87.7% and 80.2%, respectively). The prevalence of reported ACEs varied with the age of the respondent, with adolescents reporting much higher rates of exposure to violence, physical and sexual abuse than are reported retrospectively or by caregivers. This variation may partly reflect actual changes in circumstances with maturation, but may be influenced by developmental stage and issues of memory, cognition and emotional state more than has been considered in previous analyses. More research, across disciplines, is needed to understand these processes and their effect on recall. Long-term prospective studies are critical for this purpose. In conclusion, methodological research that uses a range of information sources to establish the reliability and validity of both retrospective and prospective reports ‒ recognizing that the two approaches may fundamentally answer different questions ‒ should be encouraged.

## Introduction

Made familiar by the Centers for Disease Control and Prevention (CDC) and Kaiser Permanente’s Adverse Childhood Experiences (ACE) study [1], retrospective adult reports of childhood experiences of abuse, neglect and household dysfunction have been linked to a range of adverse social and health outcomes in later life [2–21], studied predominantly in high-income countries. Growing evidence from around the world suggests that ACEs tend to cluster together [11, 17, 22, 23]; for example, that childhood sexual abuse often occurs in the presence of other ACEs [1], and that the risk for adverse outcomes increases in a strong and graded manner as the number of ACEs increase [22, 24]. The ACE score, the total number of ACEs to which an individual reports having been exposed before the age of 18, enables one to examine the cumulative impacts of ACEs on later life outcomes.

In recent years a number of South African studies have assessed the impact of childhood adversity on health and wellbeing [25, 26], including one longitudinal prospective study of cumulative adverse childhood experiences [27]. The associations between exposure to adverse childhood experiences and negative outcomes follows a pattern similar to other countries. Since the inception of its democracy more than 20 years ago, South Africa has transitioned rapidly from a setting characterized by poverty, underdeveloped infrastructure and limited resources. Yet high levels of inequality and unemployment, along with a generalized HIV epidemic adding another dimension to the experience of adversity [10, 16, 25], present conditions where exposure to multiple and concurrent adverse childhood experiences is prevalent around the country [27].

The few long-term studies that have been conducted in low- or middle-income countries (LMICs) tend to examine associations between retrospectively reported single childhood adversities and outcomes. In countries that participated in the Global School-based Student Health survey in 2003/2004, specifically Namibia, Swaziland, Uganda, Zambia and Zimbabwe, associations were found between 12-month retrospective reports of exposure to physical and sexual violence with mental health, suicide ideation, substance use, multiple sex partners and a history of sexually transmitted infection among 13-15-year-olds [28]. Other studies have linked reports of early adversity to personality and current major depressive disorders in Togo [29]; a range of sexual risk behaviours, alcohol and drug use, and intimate partner violence in South Africa, Tanzania and Zimbabwe [16]; and elevated likelihood of adult substance use disorders in Nigeria [13]. In South Africa, exposures to adverse experiences in early life have been associated with a number of poor adolescent and adult outcomes, including HIV risk [10], methamphetamine use [12], psychological distress [30], the perpetration of both non-partner and partner rape [26], and increased risk of psychiatric disorder [17].

There are some prospective studies linking exposure to adverse childhood experiences to social and health outcomes such as psychological, behavioural, and academic problems in adolescence [31]; HIV risk behaviour at age 14 [32]; obesity and type 2 diabetes [33]; mental health [34]; premature mortality [35]; chronic pain [36]; and age-related disease [37]. Most often this prospective data comes in the form of school records, which are frequently incomplete and focus on a small set of ACE variables, or historical court and child protection service records which are typically available when cases are extreme [38, 39]. There are far fewer prospective studies of adverse childhood experiences than it may appear since multiple publications using prospective data is often from a single study, as with the 1958 British birth cohort [32, 33, 35, 36, 40]. We could find no prospective or quasi-prospective studies on adverse childhood experiences and later life outcomes located in LMICs. In their 2012 meta-analysis, Varese and colleagues [41] found eight prospective studies linking childhood adversities to psychosis from the Netherlands (3), the United Kingdom (2), Finland (1), Germany (1) and Australia (1).

The reliance on retrospective recall raises questions about the extent to which reports are valid (accurate), reliable (consistent) and free from bias relevant to the hypothesis at hand. Additional methodological challenges include the possibility of confounding factors accounting for both early adverse experiences and later outcomes examined. Validity of reports depends on a number of factors, such as memory, cognitive function at the time the event occurred, and subsequent life experiences that may change a person’s outlook [42–44]. For instance, people who experience adverse health and social outcomes in later life may be more likely to recall and/or report having experienced adverse experiences during childhood [19]. Inconsistencies affecting the reliability of retrospective responses can occur for a number of reasons. Apart from deficits in memory due to a lapse in time, repression of memories may result from stressful events experienced [45]. Recall is also altered by subsequent events, whether experiences at the time or later were discussed with others or overheard, and if help or treatment was sought. Rothman and Greenland [46] propose that some of these factors might lead to misclassification of exposed individuals as unexposed, leading to a downward bias of the association between ACEs and various outcomes, a finding also reported by others [4, 47]. In contrast, the dilemma of false-positives or over-reporting is virtually impossible to establish [48].

Some research has been conducted to ascertain the reliability and validity of retrospective ACE reports. In terms of validity, it has been found that even where childhood sexual abuse has been documented, retrospective recall, even in young adulthood, can be low [49, 50]. The validity of retrospective reports is difficult to confirm [51], but establishing reliability over time of retrospectively reported adverse childhood experiences is a more manageable task. This has been done by examining the reliability of reports using a test-retest paradigm where the same respondents are questioned on two occasions [52–63]; assessing reliability using two separate measures of adversity [64, 65]; and looking at the concordance or corroboration between two different report sources [66]. A further limitation is that most studies examine the reliability of reports on only one or two adverse experiences. Fewer studies assess reliability over time of a range of childhood experiences [61].

Studies comparing the prevalence of reported adverse childhood experiences using historical prospective data such as court records and retrospective reports have found substantial under-reporting in the former [67]. Few direct comparisons of prospective and retrospective data on childhood adversities and their consequences in a single sample have been conducted. In four studies comparing documented records of child sexual and physical abuse and neglect [68–70] and child hospitalization [71] significant associations between childhood adversity and negative outcomes were found when retrospective self-reports, but not prospective documented records, were analysed.

The aim of this analysis is to use the opportunity afforded by the Birth to Twenty Plus (Bt20+) data over 22 years to explore levels of agreement and concordance in prospective reporting of ACEs from children and caregivers at different time points with retrospective reports in young adulthood.

## Methods

### Study design and participants

Ethics clearance was obtained from the Witwatersrand University Committee for Research on Human Subjects (protocol number: M140726). The Bt20+ study began as Birth to Ten, a birth cohort study in Soweto-Johannesburg with the objective of tracking a group of urban children in South Africa at a time of very significant political, social, demographic and health transitions, born as they were just weeks after Nelson Mandela’s release from prison. Extended to Bt20+, the sample consists of all singleton children born to mothers who were residents of Soweto-Johannesburg in the 7-week enrolment window and who remained in the area up until the child reached 6 months of age. The Bt20+ cohort is now 26 years old, and includes the third generation of children born to the original cohort. A detailed description of the study, its birth cohort and participants is published elsewhere [72]. The sample analysed in this paper comprises 1595 participants who were surveyed at the 21-22-year data collection point, when they provided retrospective data on adverse childhood experiences, and prospectively throughout the cohort study. Prospective reports of ACEs from parents and children were recorded at six time points across childhood and adolescence. Written informed consent was obtained from parents and guardians of all children included in the study on behalf of parents and their children. Informed assent, and later consent at the appropriate age, was obtained from children for their participation in the cohort. Since a child may not be raised by a biological parent for a number of reasons, the term *caregiver* will be used to refer to the primary caregiver of the child.

## Measures

### Adverse childhood experiences

Bt20+ collected data on a wide range of topics including variables related to adverse childhood experiences, initially from caregivers and subsequently from the Bt20+ respondents themselves. These variables include exposure to crime and violence, experiences of emotional, sexual and physical violence, poverty, family dysfunction and more. Adverse childhood experiences are defined in this study, in the same way as in the original ACE study: as physical abuse, sexual abuse, emotional abuse, physical or emotional neglect, and household dysfunction in the form of experience of divorce or parental separation, exposure to intimate partner violence (IPV), experience of living with a chronically ill or disabled individual or an individual with substance abuse problems, parental death, household legal trouble, and chronic household unemployment.

Table 1 shows the child’s age at which these variables were assessed. At Bt20+ child age five, seven and 11 years the caregiver was asked a number of ACE questions relating to her child. Between 11 and 18 years of age, the child responded to a number of ACE questions. The caregiver and adolescent reports are regarded as prospective reports of ACEs. At the 22 year data collection wave, a retrospective report on adverse experiences during the first 18 years of life was obtained from Bt20+ respondents through a set of questions modelled on the CDC/Kaiser Permanente ACE questionnaire. S1 Text lists the ACEs-related survey questions used throughout the study.

**Table 1:** Child ages at time of caregiver, adolescent & young adult ACE reports.

ACE variable	Caregiver reports			Adolescent reports			Young adult report
	5	7	11	11	15	18	23
Physical abuse	●	●		●	●	●	●
Sexual abuse	●	●		●	●	●	●
Emotional abuse	●	●			●	●	●
Divorce/separation	●	●	●	●	●		●
Exposure to IPV	●	●	●		●	●	●
Household substance abuse	●	●	●				●
Serious illness or disability in the household	●	●	●	●			●
Household legal trouble	●	●	●		●	●	●
Chronic unemployment	●	●	●	●			●
Parental death		●		●	●	●	●
Death in the family/household	●	●	●	●		●	
Separation from parents	●	●	●	●			
Exposure to violence and crime	●	●	●		●	●	●

In sum, three accounts of ACEs are examined and compared: caregiver reports about the child’s environment and experiences at the time; young adolescent reports about their own environment and experiences at the time, and young adult retrospective reports about their environment and experiences in the first 18 years of life. Prospective caregiver reports and prospective adolescent reports are individually compared to retrospective young adult reports. Combining the caregiver and adolescent self-reports a prospective report across childhood is also compared to young adult retrospective reports about the same period.

### Analysis

Descriptive statistics were used to summarize caregiver and adolescent reports of exposure to ACEs at different time points as well as the prevalence of reported ACEs. Cohen’s kappa was calculated to compare item agreements between prospective caregiver and adolescent reports of ACEs with retrospective young adult reports of ACEs. Kappa examines agreement adjusting for chance and has been used in several other studies of the reliability of reports of childhood experiences [52, 61, 73, 74]. We followed Landis & Koch’s [75] classification on the strength of agreement. The percentage of item agreement between both time points, or concordance rate, was also calculated as suggested by de Mast [76] to overcome Kappa values that may be affected by the number of ‘*yes’* responses that are due to rare events or very common events resulting in artificially low kappa values. S1 Dataset contains the minimal data used in the analysis.

## Results

**Fig 1:**
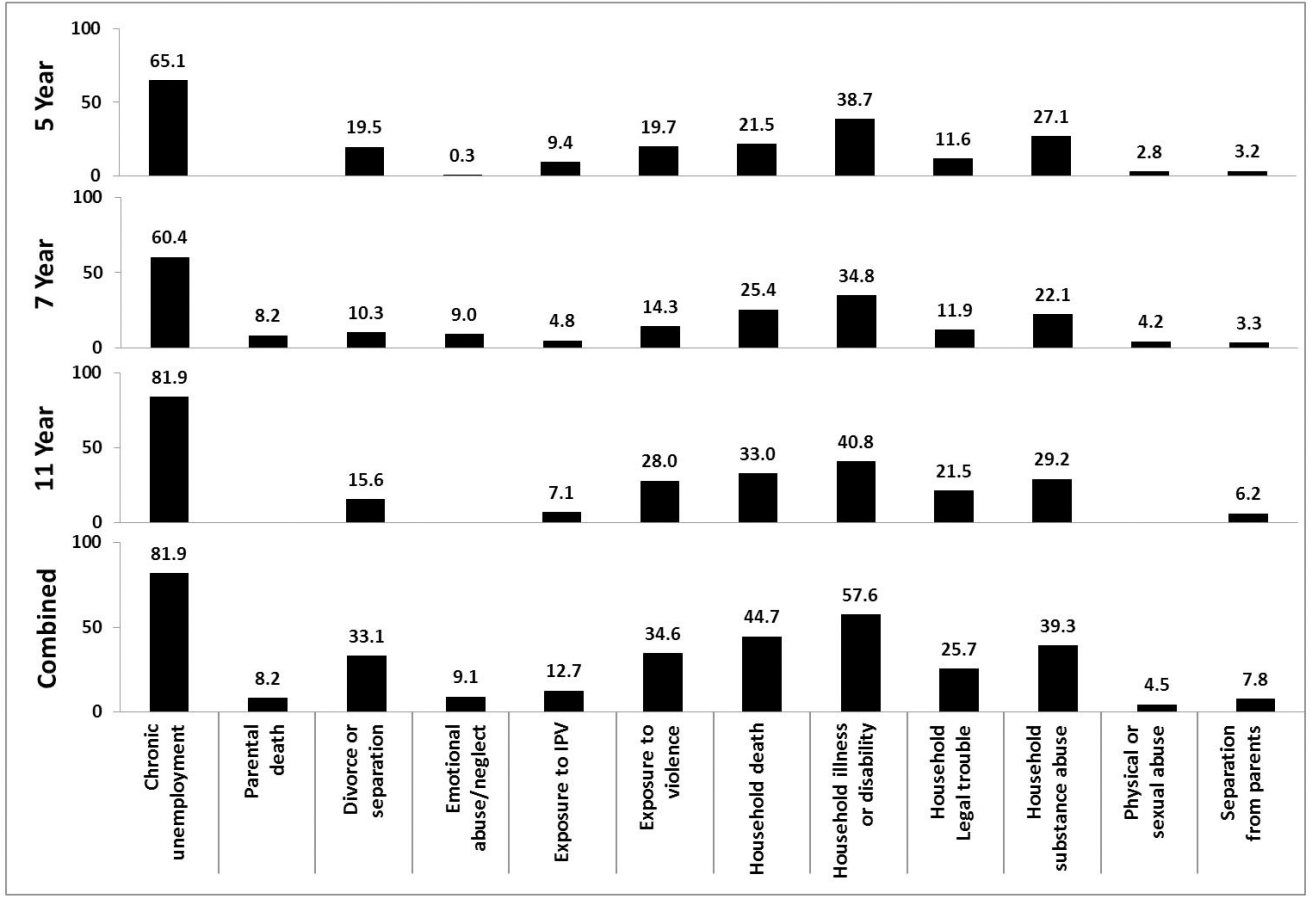
Prevalence (%) of ACEs at various child ages reported by caregiver in the first 11 years.

Fig 1 shows the prevalence of reported experience of ACEs by caregivers about children’s lives up to age 11 years. Blank spaces indicate that questions on this ACE were not asked at a particular age. A combined caregiver report is calculated as the prevalence of *ever* reporting an ACE in the 6-year period between 5 and 11 years. Divorce/separation, household substance abuse, serious illness or disability in the household, death in the household and exposure to violence and crime were reported at roughly the same rates when children were 5, 7 and 11 years old. Chronic unemployment was the most frequently reported ACE at all time points, increasing to 81.9% when the caregiver reports at child age 11 years. Combined time points show that about 82% of children younger than 11 were living in a household that reported chronic unemployment at least once.

**Fig 2:**
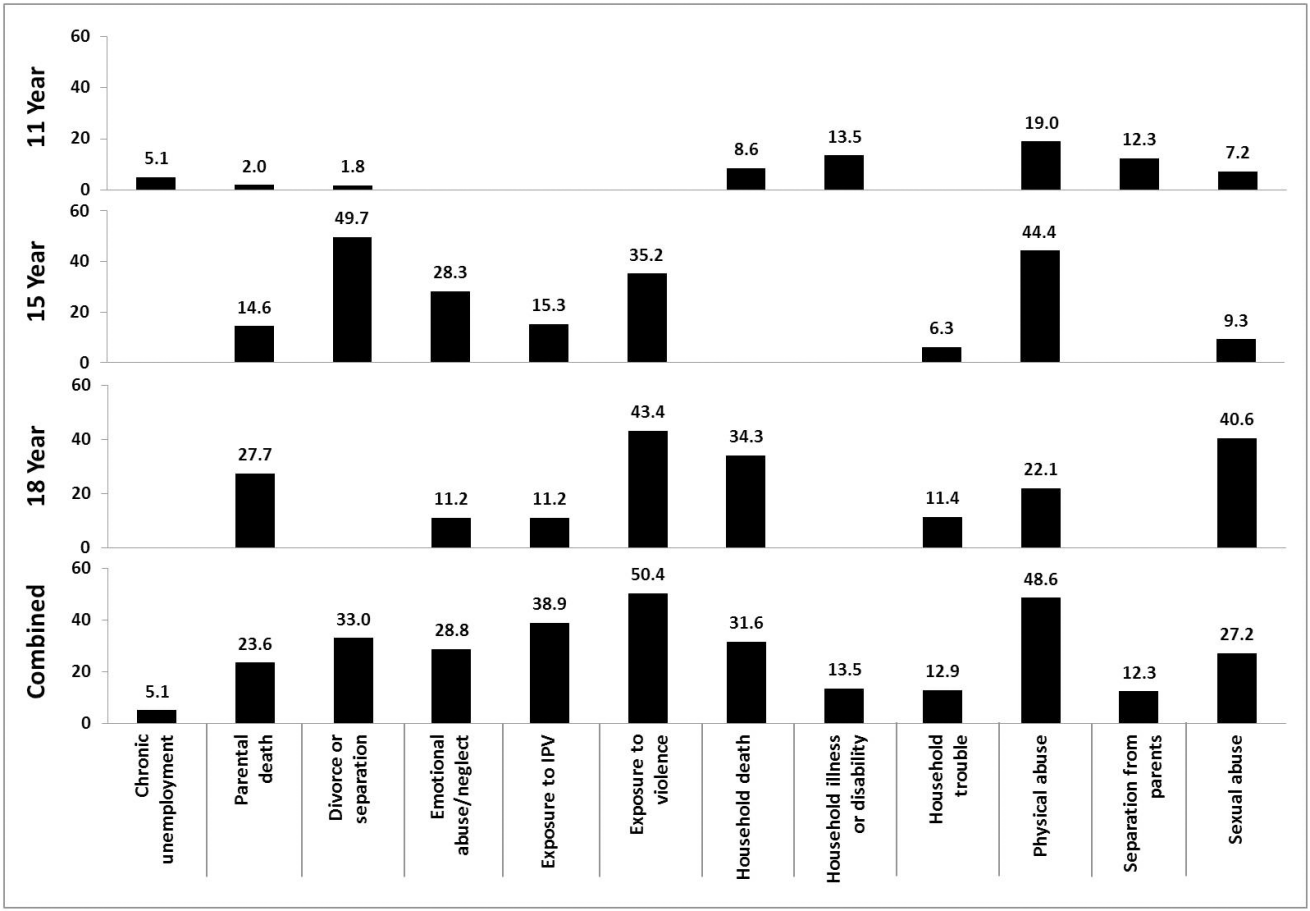
Prevalence (%) of ACEs reported by adolescents between 11 and 18 years of age.

Fig 2 looks at the average prevalence of ACEs reported by adolescents prospectively between the ages of 11 and 18. During their teenage years, young people are more likely to report the death of a parent, as well as increased exposures to violence, including sexual and physical abuse. Reports of physical abuse increase from 19.0% at 11 years to 44.4% at 15 years, decreasing to 22.1% at 18 years. Reports of sexual abuse by adolescents quadrupled from 9.3% at 15 years to 40.6% at 18 years. The combined adolescent report shows that 48.6% of adolescents report exposure to physical abuse at least once during the 11–18 year period.

**Fig 3:**
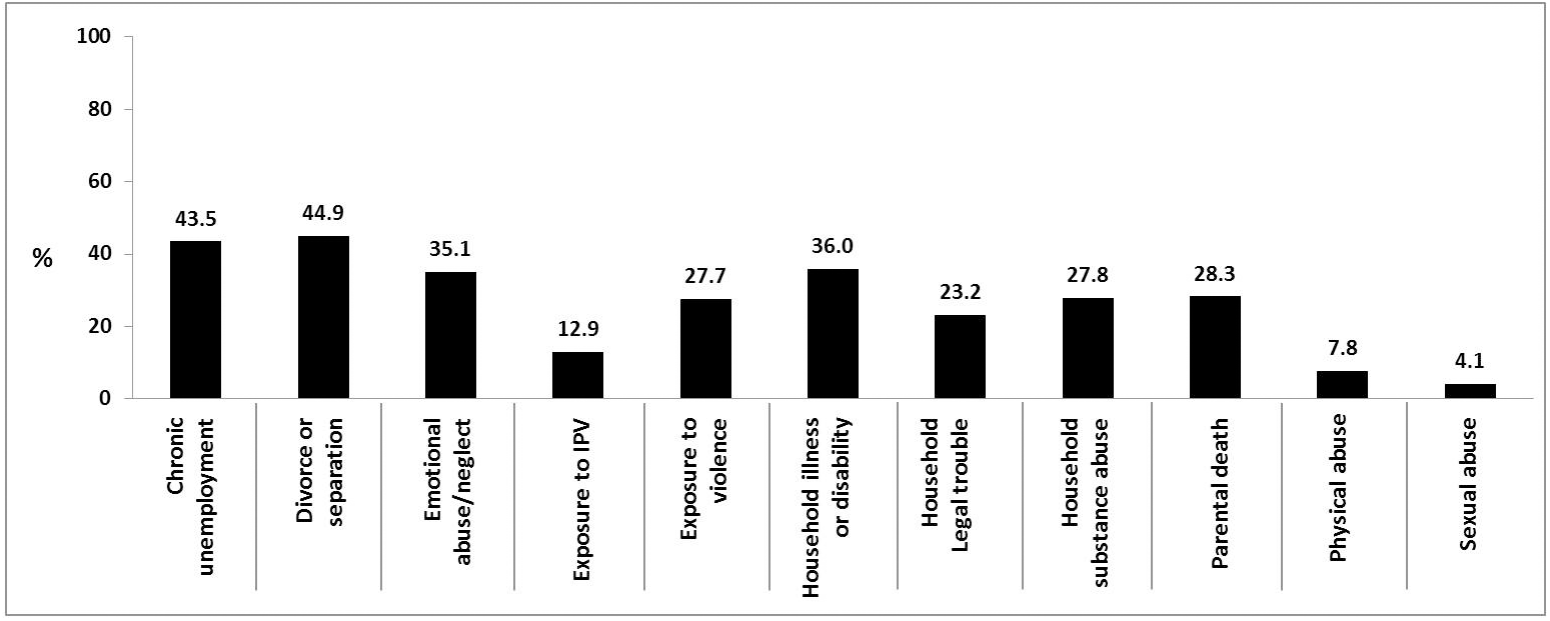
Prevalence (%) of ACEs retrospectively reported by young adults at age 21/22years.

Young adult retrospective reports of ACEs occurring before they turned 18 are shown in Fig 3. Divorce/separation and chronic unemployment in household are the most frequently reported ACEs at 44.9% and 43.5%, respectively. Retrospectively reported physical abuse is much lower ‒ at 7.8% ‒ compared to combined adolescent reporting of physical abuse at 48.6%.

**Fig 4:**
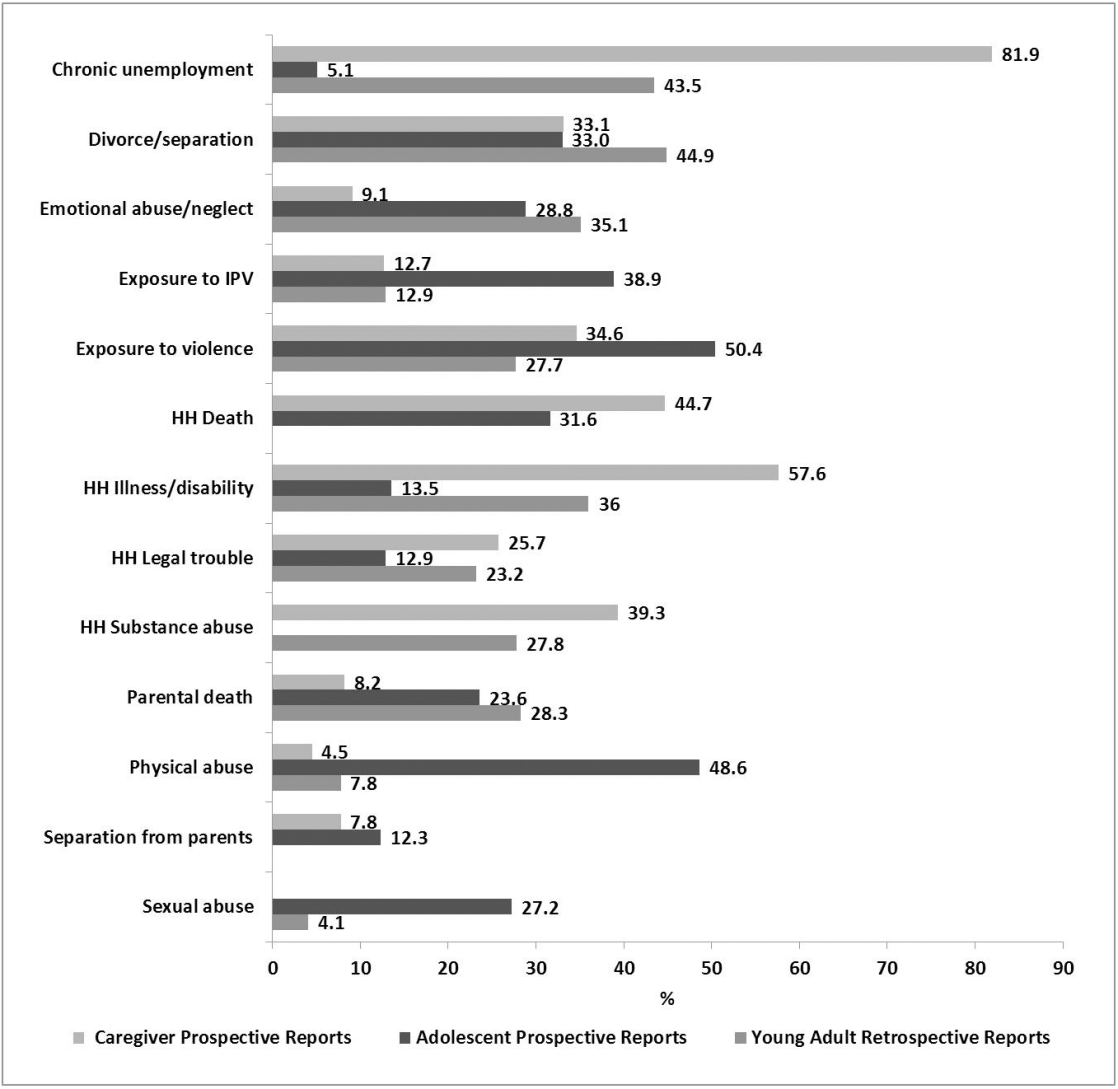
Prevalence (%) of ACEs by source and time point.

Fig 4 below shows the prevalence of reported ACEs by source ‒ caregiver, adolescent or young adult ‒ and time point ‒ prospective or retrospective. Across sources there is consistency in the reporting of exposure to violence and family instability, namely divorce/separation and child separation from parents. Adolescent prospective reports of physical and sexual abuse, exposure to IPV and more general exposure to violence are much more prominent than prospective caregiver reports or retrospective young adult reports. Caregivers report a substantial social and material burden on the household in the early years of the child’s life, with high levels of chronic unemployment, household death, illness/disability and substance abuse.

### Comparing young adult retrospective reports to caregiver and adolescent prospective reports about adverse experiences in childhood

Comparisons are made to determine the levels of agreement between prospective caregiver reports about childhood and prospective adolescent self-reports with retrospective reports by young adults. Levels of agreement between combined prospective caregiver and adolescent reports of ACEs compared to retrospective young adult reports of ACEs were also examined using Cohen’s kappa (Table 2). The concordance rates reflect total agreement on an ACE whether reported present or absent at both time points or by both sources.

**Table 2:** Level of agreement between ratings for prospective caregiver and prospective adolescent reports compared to retrospective young adult reports of adverse childhood experiences.

ACE Variable	Cell frequencies				k	Conc. Rate (%)
	N1/N2	N1/Y2	Y1/N2	Y1/Y2		
**Combined prospective caregiver and adolescent reporting (1) compared to retrospective young adult reporting (2)**						
Physical abuse	683	36	772	86	.05^**^	48.8
Sexual abuse	941	34	517	29	.02	63.8
Emotional abuse/neglect	691	304	328	244	.12^**^	59.7
Divorce/separation	473	187	245	397	.34^**^	66.8
Parental death	1022	187	97	251	.52^**^	81.8
Exposure to IPV	733	90	624	112	.05^*^	54.2
Exposure to violence	373	93	753	337	.08^**^	45.6
HH Substance abuse	612	185	496	235	.09^**^	55.4
Chronic unemployment	141	60	726	604	.07^**^	48.7
HH Legal trouble	805	189	394	175	.13^**^	62.7
HH Illness & disability	370	191	618	346	.02	47.0
HH Death	724	0	471	624	.51^**^	74.1
**Caregiver reporting (1) compared to retrospective young adult reporting (2)**						
Physical abuse	1116	94	63	7	.02	87.7
Sexual abuse	1127	49	59	3	.01	91.0
Emotional abuse/neglect	764	399	69	65	.07^**^	64.0
Divorce/separation	510	288	179	265	.22^**^	62.4
Parental death	872	294	40	78	.21^**^	74.0
Exposure to IPV	1118	132	158	53	.15^**^	80.2
Exposure to violence	671	242	396	161	.03	56.6
HH Substance abuse	642	197	435	212	.11^**^	57.5
Chronic unemployment	111	48	725	599	.05^**^	47.9
HH Legal trouble	835	221	301	127	.09^**^	64.8
HH Illness & disability	367	190	590	332	.02	47.3
HH Death	509	244	443	229	.02	51.8
**Adolescent reporting (1) compared to retrospective young adult reporting (2)**						
Physical abuse	709	38	736	82	.05^**^	50.5
Sexual abuse	968	35	475	28	.01	66.1
Emotional abuse/neglect	724	340	271	196	.11^**^	60.1
Divorce/separation	527	288	104	237	.31^**^	66.1
Parental death	1011	179	75	214	.52^**^	82.8
Exposure to IPV	807	113	532	87	.02	58.1
Exposure to violence	564	150	551	271	.02^*^	54.4
Chronic unemployment	557	396	23	27	.03	58.2
HH Legal trouble	963	260	123	69	.11^**^	73.0
HH Illness & disability	584	279	79	55	.05^*^	64.1
HH Death	1148	0	47	624	.94^**^	97.4

N1/N2 = *No* ACE reported by both; N1/Y2 = *No* reported by 1, *Yes* reported by 2

*p < .05

**p < .0001

Y1/N2 = *Yes* reported by 1; *No* reported by 2; Y1/Y2 = *Yes* reported by both

Conc. rate = concordance rate (percentage of participants with Y1/Y2 and N1/N2)

Across both combined caregiver and adolescent prospective reporting and separated caregiver and adolescent prospective reporting compared to retrospective young adult reporting there is significant agreement at *moderate* levels on parental death and household death (ranging from *k* = .51 p < .0001 to near *perfect agreement* at *k* = .94, *p* < .0001). There are *fair* levels of agreement for divorce/separation in each of the three comparisons, ranging from *k =* .22 to .34, *p <* .0001. Significant but *slight* levels of agreement are found for other ACEs across the comparisons.

**Fig 5:**
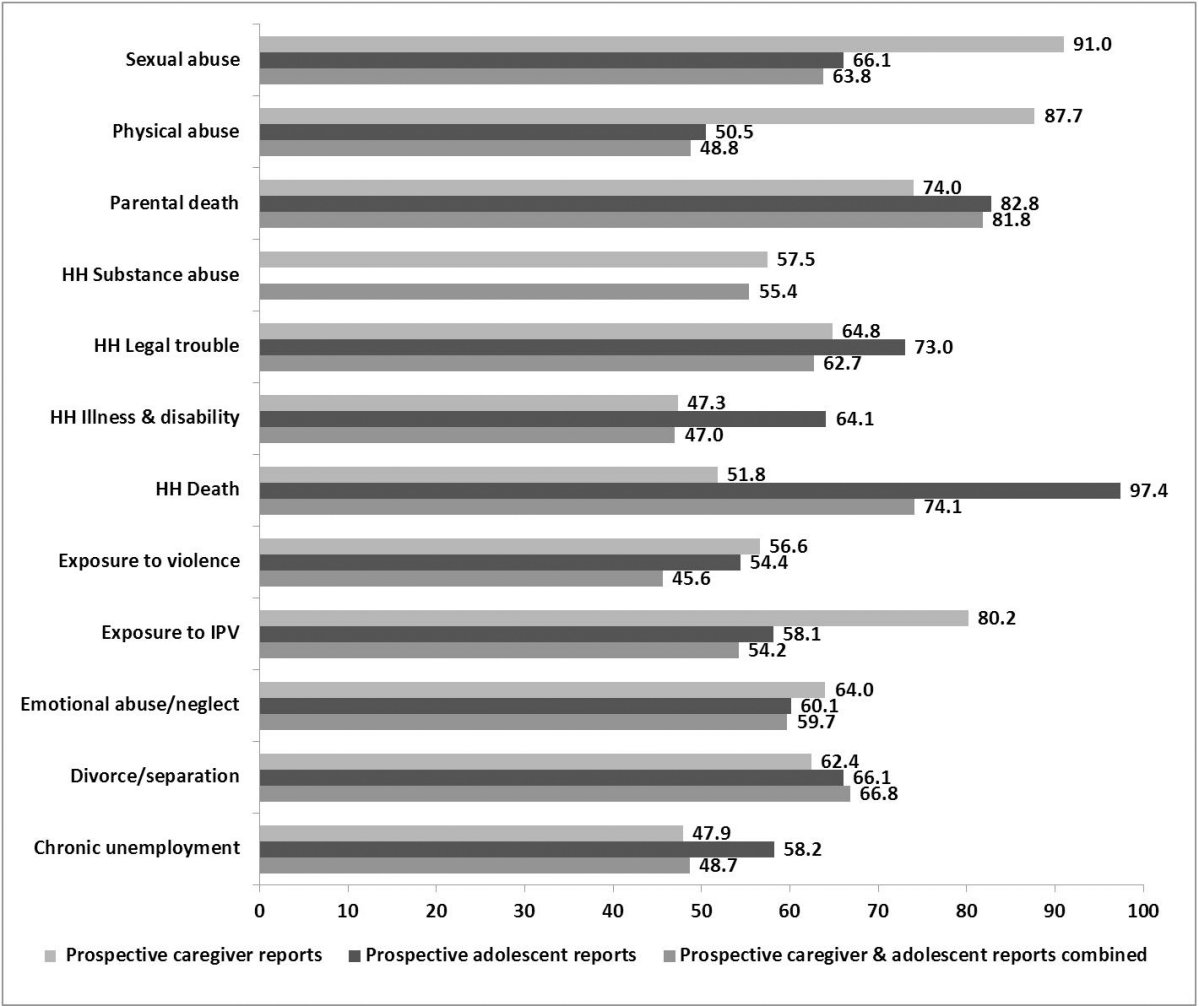
Concordance rates (%) for each ACE when prospective caregiver and prospective adolescent reports are compared to retrospective young adult reports, separately and combined.

Fig 5 illustrates the concordance rates by ACE when comparisons between accounts are seen side by side. For ACEs such as parental death, household substance abuse, household legal trouble, exposure to violence, emotional abuse/neglect, chronic unemployment, and divorce/separation the concordance between the three reports remains relatively consistent. The highest concordance rate (97.4%) is found between prospective adolescent reports and retrospective young adult reports on household death. High concordance is also seen between prospective caregiver reports and retrospective young adult reports on sexual and physical abuse and exposure to IPV (91.0%, 87.7% and 80.2%, respectively).

To understand how respondent source may play a role in reporting of ACEs, kappa values and concordance rates were calculated for ACEs that were reported on at the same time point for both caregiver and adolescent (at child age 11). Table 3 shows the levels of agreement for caregiver reported ACEs at age 11 and adolescent reported ACEs at year 11.

**Table 3:** Level of agreement between ratings for prospective caregiver reports (1) compared to prospective adolescent reports (2) of adverse childhood events at age 11.

ACE Variable	Cell frequencies				k	Conc. Rate (%)
	N1/N2	N1/Y2	Y1/N2	Y1/Y2		
Household illness/disability	549	79	370	63	.02^*^	57.7
Divorce or separation	877	14	163	3	.00^*^	83.2
Household unemployment	151	5	854	46	.01^*^	18.7
Household death	639	58	334	28	.01^*^	63.1
Child separation	888	116	47	9	.03^*^	85.1

N1/N2 = *No* ACE reported by both; N1/Y2 = *No* reported by 1, *Yes* reported by 2

*p < .05

Y1/N2 = *Yes* reported by 1, *No* reported by 2; Y1/Y2 = *Yes* reported by both

Conc. rate = concordance rate (percentage of participants with Y/Y and N/N)

Despite very low kappa values, concordance rates for reported divorce/separation and child separation are high at 83.2% and 85.1%, respectively, with both the caregiver and the adolescent reporting low levels of divorce/separation and prolonged child separation. In comparison, concordance on chronic unemployment in the household is low at 18.7%. About 4.8% of adolescents report chronic unemployment in the household compared to 80.1% of caregivers at the same time.

## Discussion

The aim of this study was to explore the levels of agreement between three accounts of reporting on ACEs ‒ prospective caregiver reports on the period between 5 and 11 years in a child’s life, prospective adolescent reports on the period between 11 and 18 years and retrospective young adult reports of experiences before the age of 18 years. In summary, we found that there was little overall agreement between combined or separate prospective accounts and retrospective accounts of childhood experiences, with a few exceptions that are described below.

### The prevalence of reported ACEs

Overall the prevalence of individual reported ACEs was fairly high, naturally increasing by age ‒‒ older children were more likely to report an ACE—with high rates of poverty reflected in the pervasiveness of unemployment at a rate of 81.9% at its highest, a finding echoed by an analysis of the prevalence of ACEs among children in a nationally representative US sample [77]. In addition to the economic hardship, the authors report high rates of exposure to violence and divorce or separation, similar to the current study’s findings. At a national level, 46% of children in the US [77] and 46.4% of participants in an English sample [78] report at least one ACE, compared to 92% in retrospective reporting and 98.9% in prospective reporting in this study. In other developing countries, similar high prevalence rates of reported ACEs are found. A retrospective study of young people in Russia estimated that 84.6% of respondents reported at least one ACE [79], and in a Brazilian birth cohort, 85% of adolescents reported at least a single ACE [80].

Depending on the type and measure of ACEs used, the timing of measurement and source, studies assessing the consistency of reporting of ACEs over time have found divergent results [51, 52, 54, 81]. There are substantial differences in the prevalence of reported ACEs across the three accounts assessed in this study. There are fairly consistent rates of reported ACEs across the three time points within the prospective caregiver reports, with less consistency in the prevalence of ACEs reported over the adolescent period. The prevalence of ACEs in adolescent reports tends to increase substantially around the 15-year period, particularly reports of physical and sexual abuse, and then decrease after the 18-year period. The prevalence of retrospectively reported ACEs by young adults is similar to that of prospective caregiver reports, particularly with regard to ACEs in the home environment such as chronic illness/disability, substance abuse, legal trouble, divorce/separation, and chronic unemployment in the household, and even extending to their own experiences of physical and sexual abuse.

Adolescents prospectively report much higher rates of exposure to violence, physical and sexual abuse than are reported retrospectively or by caregivers. As they enter secondary school and their environment expands to include peers, the range of experiences open to adolescents is greater which may explain these increases. In the adolescent reporting period, non-consensual petting and/or oral sex, in addition to penetration, are explicitly included in the operational definition of sexual abuse. As a developmental period, adolescence is also characterized by some level of egocentrism, perhaps making them acutely conscious of the events in their own lives and with a heightened perception of the severity of experiences. Research also suggests that memory is generally enhanced in adolescence and early adulthood [82], leaving adolescents less likely to forget negative experiences [83].

### Levels of agreement across time

Overall, a combined prospective account of ACEs showed only *slight* levels of agreement with retrospective young adult reports. Seventy five percent of kappa values fell within the *slight* agreement range, 8% had *fair* agreement, and 17% had *moderate* agreement. When comparing prospective caregiver reports to retrospective young adult reports, 83% of kappa values represented *slight* agreement and 17% *fair* agreement. Prospective adolescent reports, compared to retrospective young adult reports, yielded 73% *slight* agreement; 9% *fair*, *moderate* and *near perfect* agreement, respectively. In all comparisons, the highest levels of agreement were found on household death and parental death. Yancura and colleagues found similar results noting that specific events such as deaths in the family and parental separation tended to have higher kappa values than other experiences [61]. The lowest levels of agreement are found in comparisons between prospective caregiver reports of ACEs and retrospective young adult reports of ACEs. One possible reason for this is that caregiver prospective reporting covered early to middle childhood, ending when the child was 11 years of age; the years of adolescence following this period are likely to include a larger range of experiences and greater opportunity for ACEs to occur.

Concordance rates of the different ACEs across the three comparisons mirrored agreement levels with the exceptions of physical and sexual abuse and exposure to IPV in the comparison between prospective caregiver reports and retrospective young adult reports. This could be as a result of the differences in the prevalence of reported physical and sexual abuse and exposure to IPV that increase in adolescence but is not retrospectively reported on in young adulthood. Despite the low kappa values, these high levels of concordance could be due to low endorsement rates or the rarity of the event compared to other ACEs. The concordance rates for each ACE when prospective caregiver and prospective adolescent reports are compared to retrospective young adult reports, separately and combined, are fairly consistent. This suggests that the nature of the ACE will influence how it is reported, over and above timing and source issues. Apart from a few individual ACEs ‒ sexual abuse, physical abuse and exposure to IPV in the prospective caregiver report and illness/disability and household death in the prospective adolescent report ‒ the prospective accounts, separately or combined, do not appear to be highly concordant with the retrospective young adult account. This finding might have been anticipated, given that a study examining retrospective reports of childhood abuse just three years apart ‒ at 18 and 21 years old ‒ found substantial unreliability between reports [84].

### Levels of agreement across source

Comparing prospective caregiver reports over the first 11 years of a child’s life to retrospective self-reports in young adulthood is precarious in that the period of adolescence is unaccounted for. What may appear to be over-reporting in retrospective reports may simply represent events experienced during the adolescent period. But caregiver reports on the experiences in the early years of a child’s life are also useful in understanding the impact of the early environment on later life, regardless of later recall, when confounding factors can be controlled for. In the first 11 years of the child’s life caregivers report a large burden of care at the household level with high levels of substance abuse, legal trouble, chronic unemployment, chronic illness and disability, and family death. Children and adolescents may not always be aware of the level of adversity or the subsequent strain put on caregivers. In this analysis there were overall low levels of agreement between the different ACEs reported prospectively by caregivers when compared to young adults’ retrospective reports, with the exception of physical and sexual abuse and exposure to IPV which showed high levels of concordance. A study on prospective mother reports and retrospective adolescent reports found similar results of moderate agreement when looking at physical abuse [85].

When comparing levels of agreement and concordance rates on the ACEs that were reported at age 11 by both the caregiver and the adolescent, the results similarly have low kappa values. There is high concordance between caregiver and child on divorce/separation and child separation. Both caregiver and child more or less agree on relatively low levels (absence) of divorce/separation in the home, and the presence of prolonged child separation. Findings in this study suggest that there is some concordance on specific ACEs whether or not they are reported as present or absent. One study found that, when comparing mother and offspring accounts of a range of adverse experiences, the two accounts tended to correspond when the adversity was absent [66]. The lowest concordance is found on chronic unemployment with adolescents reporting much lower levels of chronic unemployment in the household than caregivers at the same time. This raises issues around the type of ACEs different sources are able to report on; younger adolescents may be unaware of financial issues and the socio-economic status of the household in early childhood. Still, little research has been conducted on parents’, particularly mothers’, ability to provide a reliable account of their children’s experiences in childhood. For a number of reasons parents may intentionally or unintentionally minimize adverse events in a child’s life [86]. A study looking at mother and offspring retrospective reports of a range of childhood adversities found that mothers tend to under-report the frequency and severity of adverse events in their offspring’s childhood [66]. In a similar study, Henry and colleagues found overall low levels of agreement between prospective mother reports collected in a birth cohort and retrospective reports collected when the respondents were 18 [87]. Agreement was higher for more objective experiences such as residential moves, but much lower for psychosocial and family processes. In a study with a larger gap between reports, Offer and colleagues compared adolescent self-reports and retrospective reports at approximate age 48 and found significant differences between what adults remember about adolescence and what was reported in adolescence [88].

Overall the findings in this study suggest unreliability when prospective reports from longitudinal data are compared to retrospective reports. While concluding that retrospective reports in adult life of adverse experiences in childhood are sufficiently valid, a review of the evidence cautions that the recall of experiences that are open to a wide degree of interpretation and rely substantially on judgment are less satisfactorily reported than those that are linked to serious abuse, neglect and conflict [51]. Issues around the design of assessments used to elicit information about sensitive childhood experiences also affect the reliability of reports. In two studies that assessed childhood abuse using different measures, fairly high rates of agreement were found [64, 65]; and through examining longitudinal data on 46 childhood experiences, Yancura and Aldwin conclude that retrospective reports may be reliable subject to well-designed assessments [61]. Examining the prospective prevalence of different reported ACEs across a number of time points shows variation in the experiences of children. It is difficult to know to what extent this variation reflects actual changes in circumstances or perceptions at different time periods. Prospective reporting may very much depend on current state or mood and retrospective reporting on disposition and life outcomes. Whether an experience becomes part of an individual’s life story, to be reported on retrospectively, depends on a number of factors both at the time of the experience and in the years following it. Research suggests that these life stories are not shaped until the post-adolescent years [89], and may not be stable until middle age [90]. Another view is that retrospective and prospective approaches address fundamentally different questions. While a prospective design examines what proportion of children exposed to adverse experiences go on to develop negative outcomes, a retrospective design assesses what proportion of individuals presenting negative outcomes report exposure to adverse childhood experiences [68]. With this in mind, it is less an issue of which design is more valid or reliable, but which is better suited to the particular inquiry.

### Study limitations

One limitation in the data is that questions probing adverse childhood experiences throughout the study were not phrased in exactly the same way at every wave of data collection. The ability to analyse agreement between sources of reporting was also limited given that there was overlap with caregiver and adolescent reports of ACEs on only a few variables at the 11 year time point.

## Conclusion

As family structures change and new environments are open to children, their experiences and their understanding of these experiences are altered. Well-designed assessments of prospective and retrospective childhood experiences help to closely capture an account of what children experience over a given period. But our individual awareness, understanding and state during and after life events play a large role in what is recalled or reported. Alluding to a quote by Offer and colleagues [88], retrospective reports may be considered “existential reconstructions” of childhood, subject to a life story that fluctuates over the life course. The challenge is to identify which account of a life story has bearing on future outcomes, and to appreciate that the level of validity and reliability of an account depends, to some extent, on the purpose of inquiry.

Overall, South African children are exposed to a large number of different ACEs throughout childhood. Their retrospective recall of these experiences in young adulthood differs substantially from what is reported prospectively. In addition to more research on the reliability and validity of different reporting methods, multidisciplinary research is needed to explore how processes of memory formation and stress responses to physical and socioemotional context, particularly in the adolescent brain, affect the perception and recall of experiences located in childhood. Both prospective and retrospective accounts of adverse childhood experiences should be understood within these parameters, and used to answer research questions appropriate to their function. Retrospective reports may be valuable in eliciting information about experiences that remain with us after certain periods of time while prospective reports may be critical for understanding the mechanisms that determine health and wellbeing outcomes based on contemporaneous experiences, regardless of later recall.

## Supporting information

S1 Text Survey questions used for data collection. (PDF)

S1 Dataset Minimal ACEs dataset. (SAV)
