# Supplementary material for: An analysis of retrospective and repeat prospective reports of adverse childhood experiences from the South African Birth to Twenty Plus cohort
Source: PLoS One. 2017 Jul 26;12(7):e0181522. doi: 10.1371/journal.pone.0181522 (PMC5528894; doi:10.1371/journal.pone.0181522)
Supplement: S1 Text — (PDF) [file pone.0181522.s001.pdf]

BIRTH TO TWENTY PLUS: ADVERSE CHILDHOOD EVENTS-SPECIFIC SURVEY QUESTIONS

Please note, the survey sections below relate to data analysed and presented in the paper “An Analysis of Retrospective and Repeat Prospective Reports of Adverse Childhood Experiences from the South African Birth to Twenty Plus Cohort” and are not inclusive of all data collected during the life of the study.

PARTICIPATION IN STUDY CONSENT

I agree to myself being a participant in Birth to Twenty Plus study
The goals and methods of the study are clear to me.
I understand that the study will involve interviews. All the details and purposes of this study have been explained to me. I understand that I have the right to refuse to participate in the study.

I agree to participation in Birth to Twenty Plus study on condition that:

1. I can withdraw from the study at any time voluntarily and that no adverse consequences will follow on withdrawal from the study.

2. I have the right not to answer any or all questions posed in the interviews and not to participate in any or all of the procedures / assessments.

3. The University of the Witwatersrand Human Ethics committee has approved the study protocol and procedures.

4. All results will be treated with the strictest confidentiality.

5. Only group results, and not my individual results, will be published in scientific journals and in the media.

6. The study scientific team are committed to treating participants with respect and privacy through interviews conducted in private and follow-up counselling available on request.

7. I will receive a referral note to a health service if any result is out of the normal range or a problem is detected in the course of the study.

PARTICIPANT:

Printed Name | **Signature / Mark or Thumbprint** | **Date and Time**

RESEARCH ASSISTANT:

Printed Name | **Signature** | **Date and Time**

SURVEY CONSENT

I agree to myself being a participant in the study
The goals and methods of the study are clear to me.
I understand that the study will involve interviews. All the details and purposes of this study have been explained to me. I understand that I have the right to refuse to participate in the study.

I agree to participation in the study on the condition that:

1. I can withdraw from the study at any time voluntarily and that no adverse consequences will follow on withdrawal from the study.

2. I have the right not to answer any or all questions posed in the interviews and not to participate in any or all of the procedures / assessments.

3. The University of the Witwatersrand Human Ethics committee has approved the study protocol and procedures.

4. All results will be treated with the strictest confidentiality.

5. Only group results, and not my individual results, will be published in scientific journals and in the media.

6. The study scientific team are committed to treating participants with respect and privacy through interviews conducted in private and follow-up counselling available on request.

7. I will receive a referral note to a health service if any result is out of the normal range or a problem is detected in the course of the study.

PARTICIPANT

**Printed Name**	**Signature / Mark or Thumbprint**	**Date and Time**

RESEARCH ASSISTANT:

**Printed Name**	**Signature**	**Date and Time**

YOUNG ADULT YEAR 22 QUESTIONS

SECTION 1: Education, Employment and Job Search

Education

The first few questions ask about your experiences with schooling and education.

1. Have you successfully completed grade 12 (passed matric, obtained matric certificate?

Yes (Skip to Q4)	No

2. If no, what is your highest grade successfully completed? (tick one)

Grade 5 or lower	Grade 6	Grade 7	Grade 8	Grade 9	Grade 10	Grade 11

3. Are you **currently** enrolled full time at high school?

Yes (Skip to Q6)	No

4. Are you **currently** doing any studies, or registered with any educational institution (including training through employment or correspondence)?

Yes, full time (Skip to Q6)	Yes, part time (Skip to Q6)	No

5. If no, please tell us the main reason that you are not **currently** registered? (**Do NOT read out options;** tick **one** only)

**Reason**	**Tick one**	**Code**
Financial constraints		1
Family responsibilities (i.e. Pregnant or Looking after children/family/relatives)		2
Completed educational goals		3
Employed		4
Taking a planned break from my studies		5
Results not good enough		6
Other (specify)		7

6. During the past six months, have you been doing any studies, or attempted to enrol for any studies?

Yes<br>(Answer the questions below)	No<br>(Skip to Q7)

Has the following happened to you in the past 6 months? (**answer all questions)**

	**Yes**	**No**
a) You failed a course		
b) You had conflict with school/university/college officials		
c) You dropped out of your training programme		
d) You had financial problems paying for school, college, university, or your training programme		

If you are still in formal full time schooling, skip to the next section (Employment)

7. Since leaving formal full time schooling, have you ever done any studies or been registered with any educational institution (including any training through employment and correspondence)?

Yes	No (skip to employment section)

8. Please complete the table below for any studies other than full time high school. Please include the 5 most recent courses. Start with the current/most recent course, and then move to older courses.

	A	B	C	D	E	F		G	H	I	J	K
	Institution Name	Institution Type<br><br>**(USE KEY)**	Location of Campus (Suburb, province)	Course Name	Qualification<br><br>**(USE KEY)**	Is the course full time or part time? (As defined by the institution)		When did you start the course? **(Month & Year)**	What is the duration of the course? **(USE KEY)**	Are you still enrolled in this course? **(USE KEY)**	How is the course funded? **(USE KEY & GIVE ALL THAT APPLY)**	What was the main reason for your choice of study? **(USE KEY)**
1						FT	PT					
2						FT	PT					
3						FT	PT					
4						FT	PT					
5						FT	PT					

	**Notes**
1	
2	
3	
4	
5	

D: Early life stress

These questions refer to when you were growing up (before 18 years of age).

	**Yes**	**No**
1. Did a parent or other adult in the household **often or very often**… Swear at you, insult you, put you down, or humiliate you? **OR** Act in a way that made you afraid that you might be physically hurt?		
2. Did you **often or very often** feel that … No one in your family loved you or thought you were important or special? **OR** Your family didn't look out for each other, feel close to each other, or support each other?		
3. Did you **often or very often** feel that … You didn't have enough to eat, had to wear dirty clothes, and had no one to protect you? **OR** Your parents were too drunk or high to take care of you or take you to the doctor if you needed it?		
4. Were your parents **ever** separated or divorced? **(If the young adult's parents' were never together, please write "NOT APPLICABLE")**		
5. Was your mother or stepmother: **Often or very often** pushed, grabbed, slapped, or had something thrown at her? **OR** **Sometimes, often, or very often** kicked, bitten, hit with a fist, or hit with something hard? **OR** **Ever** repeatedly hit at least a few minutes or threatened with a gun or knife?		
6. Did you live with anyone who was a problem drinker or alcoholic or who used street drugs?		
7. Was a household member depressed or mentally ill, or did a household member attempt suicide?		
8. Did a household member go to prison?		
9. Was there anyone in your household who was chronically ill when you were a child?		
10. Was one or more of your parents/caregivers mostly unemployed during your childhood because they could not get a job?		
11. Did either of your parents/caregivers pass away before you turned 18?		

Section 2: Early Life Experiences

These questions refer to when you were growing up (before 18 years of age).

	Yes	No
1. Did a parent or other adult in the household **often or very often**… Push, grab, slap, or throw something at you? **OR** **Ever** hit you so hard that you had marks or were injured?		
2. Did an adult or person at least 5 years older than you **ever**… Touch or fondle you or have you touch their body in a sexual way? **OR** Attempt or actually have oral, anal, or vaginal intercourse with you?		

QUALITY CHECKED BY: ______ **DATE:** ______

Notes:

ADOLESCENT QUESTIONS

The SIXTH section of the questionnaire we are going to talk about VIOLENCE in your community and school

How often do the following apply to you (not on TV or in movies)	**Never**	**Once or twice**	**A few times**	**Many times**
I have heard gun shots				
I have seen somebody arrested				
I have seen drug deals				
I have seen someone being beaten up				
My house has been broken into				
I have seen somebody get stabbed				
I have seen somebody get shot				
I have seen a gun in my home				
I have seen alcohol such as beer, wine, or hard liquor in my home				
I have seen gangs in my neighbourhood				
I have seen somebody pull a gun on another person				
I have seen someone in my home get shot or stabbed				

**At school,** how often have you been:	**Never**	**Once or twice**	**A few times**	**Many times**
Hit by a student				
Hit by school staff				
Kicked or pushed by a student				
Kicked or pushed by school staff				
Badly beaten up				
Threatened with a knife or sharp weapon				
Attacked with a knife or sharp weapon				
Threatened with a gun				
Verbally or emotionally abused by a student, that is, being called names or having things said to you that make you feel bad about yourself or afraid				
Verbally or emotionally abused by school staff				
Sexually harassed by a student (unwelcome advances which continue after saying no)				
Sexually harassed by school staff				
Sexually assaulted (attacked)				
Robbed				

**In your neighbourhood,** how often have you been:	**Never**	**Once or twice**	**A few times**	**Many times**
Hit				
Kicked				
Pushed or shoved				
Badly beaten up				
Threatened with a knife or sharp weapon				
Attacked with a knife or sharp weapon				
Threatened with a gun				
Verbally or emotionally abused, that is, being called names or having things said to you that make you feel bad about yourself or afraid				
Shot at				
Sexually harassed				
Sexually assaulted (attacked)				
Robbed				

**At home, in the past,** how often have you been:	**Never**	**Once or twice**	**A few times**	**Many times**
Hit				
Kicked				
Pushed or shoved				
Badly beaten up				
Threatened with a knife or sharp weapon				
Attacked with a knife or sharp weapon				
Threatened with a gun				
Verbally or emotionally abused, that is, being called names or having things said to you that make you feel bad about yourself or afraid				
Shot at				
Sexually harassed				
Sexually assaulted (attacked)				
Robbed				

**At school,** how often have **YOU** done these things:	**Never**	**Once or twice**	**A few times**	**Many times**
Hit or kicked someone				
Pushed or shoved someone when you were angry				
Badly beaten someone up				
Threatened someone with a knife or sharp weapon				

Attacked someone with a knife or sharp weapon				
Threatened someone with a gun				
Verbally or emotionally abused someone, that is, being called names or having things said to you that make you feel bad about yourself or afraid				
Sexually harassed someone				
Robbed someone				
Been suspended from school				
Gotten into a fight after drinking or getting high				

**Outside of school ,** how often have **YOU** done these things:	**Never**	**Once or twice**	**A few times**	**Many times**
Hit or kicked someone				
Pushed or shoved someone when you were angry				
Badly beaten someone up				
Threatened someone with a knife or sharp weapon				
Attacked someone with a knife or sharp weapon				
Threatened someone with a gun				
Verbally or emotionally abused someone, that is, being called names or having things said to you that make you feel bad about yourself or afraid				
Sexually harassed someone				
Robbed someone				
Gotten into a fight after drinking or getting high				

Question 3

Have you ever been physically hurt by -

friend	**NO**	**YES**
boyfriend / girlfriend	**NO**	**YES**
peers at school	**NO**	**YES**
family	**NO**	**YES**
strangers	**NO**	**YES**
others (please specify)		

Question 2

Have you ever engaged in **foreplay** or **heavy petting** (kissing, fingering, romancing, NOT going "all the way") in the past **month (30 days)**?

NO ☐	YES ☐
**If YOU TICK (√)"NO": go to Question 5**	**If YOU TICK (√) "YES": please answer the following questions**<br>1. How old was the person you engaged with? ☐ Years<br>2. Was this something you wanted to do? NO ☐ YES ☐

Question 4

Have you ever had **SEX** (made love/ gone all the way/ penis inserted in vagina or anus)?

NO ☐	YES ☐
**If YOU TICK (√) "NO": go to Question 6**	**If YOU TICK (√) "YES": please answer the following questions**<br>1. How old were you in years when you had sex? ☐ years<br>2. How old was your first partner? ☐ years<br>3. Was this something you wanted to do? NO ☐ YES ☐<br>6. Did you make use of a male condom / rubber / female condom? NO ☐ YES ☐<br>5. Did your partner make use of a male condom / rubber / female condom? NO ☐ YES ☐<br>.

LIFE EVENTS

Please tick all appropriate events that have happened in the **past 6 months**

Family moved to a new house	
Birth of a brother or sister	
Death of a parent	
Serious illness requiring hospitalization of brother or sister	
Marriage of parent to stepparent	
Divorce of a parent	
Changed schools	
Serious illness or accident requiring hospitalization for you	
Marital separation of parents	
Increase in arguments between parents	
Serious illness or accident requiring hospitalization of parent	
Death of a close friend	
Separation from close family for 2 weeks or more	
Death of a brother or sister	
Death of a grandparent	
Brother or sister leaving home	
Loss of job by parent	

7. Have you ever had sex or been touched in private areas against your will? — No 0 Yes 1 Don't Know 2

If YES: How old was the person who did this to you ☐

What sex/gender was the person? — Male 1 Female 2

Have you ever had sex or touched another person in private areas against his/her will? — No 0 Yes 1 Don't Know 2

If YES: How old was the person you did it to? ☐

CAREGIVER QUESTIONS

23. Has your child been or have you ever suspected that your BTT child has been physically or sexually abused by somebody?

Yes=1	No=0	Not sure=2

IF YES:
Who? ____

What happened or what did you think happened?

____

____

STRESS AND VIOLENCE:

Sometimes one's life and that of one's close family, goes through periods of being very stressful. I'd like to ask you some questions about any stress you might have experienced in the last few months.

62. During the last 6 months, have you or a member of your close family been in real danger of being killed? | Yes=1 | No=0 |

63. During the last 6 months has any household member died as a result of violence in the areas where you live or work? | Yes=1 | No=0 |

64. During the last 6 months, has any household member been a victim of a violent crime (like armed robbery, you live or work? | Yes=1 | No=0 |

65. During the last six months, has any household member been a victim of a violent crime (like armed robbery, assault, rape etc)? | Yes=1 | No=0 |

66. During the last 6 months, did you witness a violent crime (e.g. murder, robbery, assault, rape)? | Yes=1 | No=0 |

67. During the last 6 months, has violence in the areas where you live or work affected your ability to obtain health care for any of your children? | Yes=1 | No=0 |

68. During the last six months, have you found that you are in so much debt that you don't know how you will repay the money? | Yes=1 | No=0 |

69. During the last six months, have you or your close family ever had too little money for basics, such as food, rent, clothes? | Yes=1 | No=0 |

70. Have you or one of your close family not been able to find a job more than six months? | Yes=1 | No=0 |

Question	Yes	No
70. Have you or one of your close family not been able to find a job more than six months?	Yes=1	No=0
71. During the last six months, have you or anyone in your close family been seriously ill?	Yes=1	No=0
72. During the last six months, did any member of your close family die?	Yes=1	No=0
73. Is there anyone in your close family with a serious disability (for example, epilepsy, mental retardation,deafness,blindness, mental illness)	Yes=1	No=0
74. Is there anyone in your close family that has a problem with drugs or alcohol?	Yes=1	No=0
75. During the last six months, have you had a break-up with your husband or partner?	Yes=1	No=0
76. During the last six months, has your husband or partner hit or beaten you?	Yes=1	No=0
77. During the last six months, have you had any serious fight or alienation from members of your family or your close neighbours?	Yes=1	No=0
78. During the last six months, have you or any member of your close family been arrested, had to go to court, or consulted a lawyer on a non-routine matter?	Yes=1	No=0
79. During the last six months, have you given help (money, accommodation etc) to close family or friends in need?	Yes=1	No=0
80. During the last six months, have you been separated unwillingly, from any of your child/ren (excluding holidays)	Yes=1	No=0
81. During the last six months, have you experienced any problems with your child or children (such as schools closing, failure at school, problem behavior, drugs etc.)	Yes=1	No=0

IF YES: Specify problem: ______________________________
